# Patient organization involvement and the challenge of securing access to treatments for rare diseases: report of a policy engagement workshop

**DOI:** 10.1186/s40900-017-0065-z

**Published:** 2017-09-04

**Authors:** Koichi Mikami, Steve Sturdy

**Affiliations:** 1 Science Interpreter Training Program, KOMEX, University of Tokyo, Tokyo, Japan; 2 Science, Technology and Innovation Studies, SSPS, University of Edinburgh, Edinburgh, UK

**Keywords:** Rare diseases, Orphan drugs, Patient organizations, Partnership, Drug development

## Abstract

**Plain English summary:**

Patients with rare diseases often help to develop new treatments for their conditions. But once developed, those treatments are sometimes priced too high for many patients to access them. We became aware that this is a problem in the course of a social science research project that examines the place of rare diseases in health policy. We therefore organized a two-day workshop to try and understand why this problem occurs and what might be done about it.

The people who participated in our workshop were: representatives of rare disease patient organizations, experts in matters of drug regulation and assessment of new health technologies, consultants involved with companies producing treatments for rare diseases, and social scientists researching related issues.

The main conclusions to emerge from the discussions were as follows:

Problems of access to treatments for rare diseases are not just due to high prices; procedures for regulating, assessing and delivering new treatments also need to be better organized. Patients and patient organizations have much to contribute to this process. However, their resources are often very limited. Consequently, more needs to be done to help them use those resources as effectively as possible. In particular, regulators and healthcare providers need to ensure that their procedures are clear and efficiently managed, so as not to waste patient organizations’ time and money. Clearer guidance is needed on what patient organizations can do to provide evidence of the effectiveness of new drugs. Insights gained in tackling rare diseases might also be applicable to common disorders. Finally, the consequences of Brexit for UK policies on rare diseases urgently need to be assessed.

**Abstract:**

Since the enactment of orphan drug legislation in the USA, Europe and several other countries, an increasing number of treatments for rare diseases have been developed and many of them been approved for marketing. However, such treatments tend to be priced very high, and access to effective treatments remains a major challenge for patients with rare diseases – despite active involvement of patients and their support organizations in various stages of basic and applied research and commercial development. In order to allow patients to benefit from treatments proved effective for their diseases, we need to better understand why this challenge persists, and what steps might be taken to address it. To that end, we organized a policy-engagement workshop, bringing together individuals and organizations with direct experience of trying to secure access to a treatment for a rare disease along with individuals with relevant expertise in regulatory and commissioning processes for new medicines. With additional input from social scientists who offered different perspectives on the value of patient involvement, the workshop aimed to initiate a dialogue among the participants about how to address the challenge in a sustainable manner. Discussions at the workshop stressed that active involvement of patients is as valuable in the regulatory and commissioning processes as in the research and development of new medicines. However, it also highlighted certain risks and costs associated with such involvement. These include the costs of adjusting to abrupt changes in regulatory and commissioning processes, and the risk of being perceived as too close to commercial interests. To optimize use of scarce resources and ensure continuing active involvement, such risks and costs need to be better managed. Participants also noted that, owing to advances in genomic technologies, common diseases are also becoming divided into rare sub-categories, which are equally eligible for orphan drug designation. Consequently, involvement of wider patient communities beyond rare disease communities will be critical for continuing discussions about patients’ involvement in regulatory and commissioning processes, and to consider how patients and their support organizations can best work with other stakeholders – including companies, regulators and policymakers – to ensure access to effective medicines.

## Background

Rare diseases are one of the areas of medicine in which patient involvement and engagement has become most deeply entrenched, in relation both to research and development and to policymaking and health service delivery. This involvement has evolved over time. The role of patient organizations in rare disease research used to be primarily *auxiliary* – financially supporting work led by medical professionals, or donating specimens required for undertaking such work. However, some rare disease patient organizations have recently been successful in assuming a more active role as *partners* to the professionals, shaping research agendas and study designs to reflect their own perspectives [1, 2]. When an international consortium for rare diseases research was launched in 2011 with the goals of developing new means of diagnosis and therapies for the diseases, it adopted a policy that research on rare diseases should involve patients and/or representatives of patient organizations in all relevant aspects of the work [3]. Their involvement is considered to be critical because patients and their carers possess different kinds of knowledge of their conditions from that of the professionals [4].

Involvement of rare disease patients and their families has been particularly successful, in part because of the unique challenges that they face due to the rarity of their diseases. In the past, many felt that that rarity led to scientists and policymakers paying too little attention to their diseases. However, the situation started to change when patients’ advocacy efforts and close working with legislators resulted in the passage of the Orphan Drug Act of 1983 in the United States [5, 6]. When consideration of similar legislation in Europe took off in the mid-1990s, medical professionals and policymakers sought to actively involve patient organizations in the process, which led to enactment of the EC Regulation on Orphan Medicinal Products in 2000 [7]. This Regulation established, at the European Medicines Agency (EMA), the Committee for Orphan Medicinal Products (COMP) – a body responsible for conferring orphan drugs designation on products intended for diagnosis, prevention, or treatment of any life-threatening or seriously debilitating disease or condition which affects no more than 5 in 10,000 people in the European Union. The Regulation also specified that the Committee should include three representatives of patient organizations as full members [8, 9]. Reflecting on the important roles that rare disease patient organizations have played in promoting policies and advancing biomedical sciences since the late twentieth century, some medical professionals consider them to be “among the most empowered groups in the health sector,” suggesting that their work can be a model for other organizations that seek better diagnostics and treatments for their diseases [10].

The active engagement of patients with rare diseases and their support organizations is now considered valuable in the commercial domain too. In the early 2010s, some families and patient organizations sought to realize that value by involving themselves directly in developing orphan drugs – in one case by founding a start-up company to develop a promising line of treatment, in another by initiating clinical trials of a drug that has shown some evidence of efficacy [11, 12]. More generally, because the low incidence of rare diseases often makes it difficult to collect clinical evidence by conventional means, such as randomized controlled trials, the EMA and COMP now encourage companies developing orphan drugs to engage with patients as early as possible so as to adequately address the unmet medical needs from the patients’ viewpoint [9]. Such involvement is often invaluable in helping companies secure approval of their products, as patients can advise on practicable means of collecting clinical data, advise on relevant criteria of clinical efficacy, and represent the human aspect of their condition to regulators.

Despite this active involvement in research and policy, however, rare disease patients often face serious challenges in securing access to treatments for their conditions. Orphan drug legislation in the United States, Europe and a few other countries offers incentives for companies to develop drugs for rare diseases, and has successfully increased the number of such drugs available on the market [13, 14]. However, manufacturers often charge very high prices for such drugs. Consequently, according to one analysis, most orphan drugs would fail to meet the standard cost-benefit criteria employed in health technology assessment (HTA) [15], and payers – be they private insurance companies or national health services – commonly question their affordability. In some cases, patients have been granted access to a treatment on an ad hoc basis – for example, an arrangement at the National Health Service (NHS) in the United Kingdom for an enzyme replacement therapy for Gaucher disease – but the social and ethical acceptability of meeting such costs through special arrangements at the expense of treatments for other conditions has been a subject of heated debate [16, 17]. Consequently, patients with seriously debilitating or life-threatening conditions may go untreated even though effective treatments are available. The situation is likely to become increasingly common in the future, as treatments are found for a growing number of rare diseases, of which more than 7000 are currently known. It will also apply increasingly to common disorders such as cancer, as they become subdivided into rare sub-categories that qualify for inclusion under orphan drug legislation. A means of ensuring access to such treatments, while avoiding unsustainable increase in public expenditure on health services, and without exacerbating the divide between those who can and cannot afford them, is therefore urgently needed [18]. The role of patients and patient organizations in developing such an approach will be crucial.

## Method

In order to better understand the reasons underlying the problem of access to treatments for rare diseases, and to consider what steps might be taken to address that problem, the authors organized a two-day workshop entitled *Rare Diseases, Patient-Private Partnership, and Access to Treatments* at the University of Edinburgh in June 2016.

The workshop was organized as part of the Wellcome Trust-funded research project Making Genomic Medicine, which aims to engage critically with key issues in modern medicine by undertaking socio-historical investigations into the development of medical genetics and genomic medicine. One such development that the project is studying is the increased prominence of rare diseases in medical research and health policy. The workshop was prompted in part by the interactions that one of the authors (KM) had with rare disease patients, representatives of rare disease organizations, and others who in one way or another have been trying to address the problem of access described above, and in part by the realization that this is a major policy issue which will have implications not just for a growing range of rare diseases, but for the sustainability of health systems more generally.

The workshop was designed as a *policy-engagement* workshop, following a model developed by the Genomics Policy and Research Forum – an experimental initiative to explore the intersections of genomic science and society, which was funded by the UK Economics and Social Research Council between 2004 and 2013 [19]. It should not be confused with a public engagement workshop. Typically, public engagement workshops involve engaging with a representative or otherwise salient segment of the public in order to ensure that their views, concerns, and critiques on a selected theme are taken into account in the development of research, service provision or policy. By contrast, the policy-engagement workshop model aims to bring together a group of stakeholders from a range of relevant sectors, including but by no means confined to relevant ‘publics’, and to provide them with a ‘safe space’ in which to start a constructive dialogue on an emerging or otherwise unresolved matter of policy. The purpose of the discussion is to try to develop a shared understanding of the problem at hand, and to identify ways in which different stakeholders might continue to work together to address that problem [20]. In effect, policy-engagement workshops are intended to encourage and initiate the kind of ‘collective puzzling’ that often underlies successful policy development, and to define and frame issues around which wider collective discussion and action might usefully be conducted [21]. As such, we find that they work best in exploring issues that have not yet attracted much notice from policy makers or other concerned groups – as for instance the workshop reported here – and are far less effective as a means of resolving established matters of disagreement. To ensure that dialogue proceeds as constructively as possible, the number of participants is usually small (experience suggests around 20–25 as a practical maximum), and participants are selected to ensure that relevant viewpoints are represented while as far as possible avoiding individuals known to be dogmatic or confrontational in their behaviour.

The workshop discussed here accordingly brought together representatives of rare disease patient organizations with other stakeholders with experience of policy, regulatory and commercial activities around access to treatments for rare diseases. Some of the participants were themselves rare disease patients or parents of children with rare diseases, but that was not a positive selection criterion so much as a reflection of the social make-up of the rare disease field itself. Participants – mainly but not solely from the UK – were selected and invited to represent a broad range of perspectives and experience of relevant issues; where appropriate, they participated in a personal capacity, representing their own personal understanding rather than the views of the organizations for which they worked or had worked. The planning and organization of the workshop was led by the authors, who are both social scientists by training with no personal stake in the issues to be discussed, beyond their academic interest in the medical and policy problems of rare diseases. In selecting participants to invite, the organizers drew on their own knowledge of the field to identify individuals with relevant knowledge and experience. We also drew on the expertise and experience of members of the advisory committee to the Making Genomic Medicine project, in particular Alastair Kent, then director of the Genetic Alliance UK and chair of the national multi-stakeholder campaign Rare Diseases UK, and John Purves, former officer of the EMA. In some instances, participants were also suggested by invitees who were themselves unable to attend.

In selecting workshop participants, we made a deliberate decision not to include representatives of patient organizations for cancer, though a growing number of orphan drugs have been developed for rare forms of cancer, and patients are likely to encounter similar problems of access to innovative treatments. Historically, however, scientific and medical understanding of rare cancers, the politics of medical research and healthcare provision for cancer, and the formation of rare cancer patient organizations, have all followed very different trajectories from the group of conditions more usually regarded as “rare diseases”. In the interests of keeping the topic of discussion within manageable bounds, we therefore decided not to address rare cancers – though there is undoubtedly much to be gained from bringing the two communities into closer correspondence in future.

Even while confining ourselves to rare diseases as usually understood, we were conscious that rare disease organizations vary enormously in size, resources, and aims, and hold disparate ideas about whether and how best to partner with one another and with researchers, healthcare providers, policy makers and pharmaceutical companies. We therefore took care to invite participants from a range of organizations of different styles and sizes. Nonetheless, the authors were acutely aware that limiting participation to a small number of pre-selected invitees and excluding certain sectors meant that the kinds of perspectives and experiences represented would inevitably be partial. However, the aim of the workshop was not to ensure that all relevant voices and publics were represented, but rather to initiate a constructive process of dialogue and reflection among key stakeholders that would raising salient issues, demonstrate the value of engagement in doing so, and thus contribute to longer-term and more inclusive processes of policy formation. To that end, invitees were sent a short briefing document outlining the key policy issues as understood by the organizers, but discussion at the workshop itself was deliberately kept as open-ended and exploratory and dialogical as possible, and took place under the Chatham House Rule – that is, with agreement that while the views expressed at the meeting might be reported, they would not be attributed to particular participants – so that participants would feel free to speak frankly [22]. In addition, a small number of social scientists working in relevant areas were also invited, whose task was to offer perspectives that might help participants to step back from their own engaged experience and to appreciate it in the larger social and institutional context within which they operate. (For a list of the workshop delegates, see Table 1).

**Table 1:** List of delegates (in alphabetical order)

Name	Relevance to the topic
Campbell, Jean	Founding member of Professional Patient Advocates in Life Sciences, a non-profit organization in the United States to support patient-advocacy professionals in pharmaceutical and biotechnology companies.
Greene, Lesley	Founder of Children Living with Inherited Metabolic Diseases (CLIMB), founding member of EURORDIS, and currently a patient-representative member (2009-) and vice chair of COMP (2012-).
Hayden, Cori	Professor of Anthropology at the University of California Berkeley, with research interest in benefit-sharing arrangements in biosciences.
Kent, Alastair	Director of Genetic Alliance UK and chair of Rare Diseases UK, which is a multi-stakeholder campaign in the United Kingdom for patients with rare diseases and all who support them.
Livingston, Heidi	Public Involvement Advisor at the National Institute for Health and Clinical Excellence (NICE) (participated in a personal capacity as an expert in patient involvement in technology appraisal processes).
Meadowcroft, Robert	Chief executive of Muscular Dystrophy UK, a charity organization supporting individuals affected by muscle-waisting conditions in the United Kingdom.
Mikami, Koichi	Research fellow in Making Genomic Medicine at the University of Edinburgh and co-organizer of the workshop.
Moreira, Tiago	Reader at Durham University with research interest in the roles of patient organizations in the organization of health care and governance of biomedicine.
Parker, Samantha	Head of Patient and Policy Affairs at biotechnology company Lysogene in France.
Pavelin, Colin	Head of Regenerative Medicine and Rare Disease Policy at the UK Department of Health (participating in a personal capacity as an expert in national policies on rare diseases in the UK).
Purves, John	Former head of the Quality of Medicines sector at the European Medicines Agency, and an honorary fellow of the Innogen Centre at the University of Edinburgh.
Roberts, Charlotte	Communications Officer of the MPS Society, a charitable organization supporting individuals, families and professionals affected by mucopolysaccharide and related diseases throughout the United Kingdom.
Schoneveld van der Linde, Maryze	Former board member of the International Pompe Association, and a founder of consultant company Patient Centered Solutions in the Netherlands.
Spink, Jayne	Chief Executive Officer of the Tuberous Sclerosis Association, a charity supporting individuals affected by tuberous sclerosis complex and their families and carers in the United Kingdom and funding research.
Spring, Rachel	Theme Coordinator at the National Institute for Health Research’s Rare Diseases Translational Research Collaboration
Sturdy, Steve	Professor of the Sociology of Medical Knowledge at the University of Edinburgh, principal investigator of the Making Genomic Medicine project, and co-organizer of the workshop.
Timmis, Oliver	Chief Executive Officer of the AKU Society, a charity organization supporting individuals affected by alkaptonuria in the United Kingdom that initiated a EU-funded consortium called DevelopAKUre program.
Upadhyaya, Sheela	Associate Director of Highly Specialised Technology program at the National Institute for Health and Clinical Excellence (participated in personal capacity as an expert in evaluation of medicines for rare diseases).

The workshop consisted of four sessions. The first three sessions followed the same format, starting with short presentations from selected delegates and then moving on to open discussion. But each session had a different focus: the first session entitled ‘Costs and Benefits of Partnership’ explored the experiences of rare disease patient organizations and their views on the challenges and opportunities in partnering with other stakeholders; the second ‘Rethinking Partnership’ looked at the social science perspectives on the types of social contract between ‘the studying’ and ‘the studied’; and the third ‘Current Terms of Partnership’ examined the intersections of patients and companies and ways of avoiding or minimizing potential conflicts of interest. The final session then aimed to reflect on the outcomes of the preceding sessions, with opening comments from four of the participants followed by general discussion.

The present report was then drafted by the authors, based on copies of the presentations, and notes and audio recordings of the discussions. The main purpose of the report was to capture and publicize the key conclusions to have emerged from those discussions, with the purpose of stimulating and informing further policy action around access to medicines for rare and other diseases. The draft report was circulated for comment to all the participants, and revised in light of their feedback, so as to ensure that the issues presented here adequately reflect the discussion rather than the academic interests of the authors. All participants agreed that the results section of this paper was a satisfactory representation of the main substance of the workshop conversations. Additionally, consent to publish a full list of delegates was obtained from all the participants; while this is not always the case for meetings held under the Chatham House Rule, we felt that it would enable readers to better appreciate what viewpoints and interests were represented and voiced in the workshop.

## Results

Although the conversation at the workshop inevitably ranged beyond the specific question of patient-company partnerships and their implications for access, discussion concentrated around the six key sets of issues presented in this section. Participants did not necessarily agree on how to address these issues – on the contrary, some topics were important precisely because they provoked disagreement – but all are clearly issues around which further discussion or action is required.

## 1. The cost of drugs is important but not the only problem

Problems of access to treatments for rare diseases are generally attributed to their high price. While this may be true for some innovative medicines, including enzyme replacement therapy and gene therapy, other problems can also be identified. In particular, patients with some rare conditions might also benefit from cheaper drugs already available on the market for other indications. However, such drugs may still need to overcome regulatory obstacles, often involving substantial delays, before they are made available to patients with rare diseases. While such drugs may be available off-label, clinicians or health care providers may be unwilling to use them in such circumstances because of potential liability risk, leaving patients without access to them.

This is not to suggest that the cost is not also a problem; on the contrary, the high price of some innovative medicines, and the fact that some patients may have to depend on them throughout their lives, causes considerable concern for both patients and healthcare providers, and can sometimes be a problem for suppliers in the long term too. In some countries, including the United States, suppliers offer subsidies to patients who cannot afford their drugs through patient support or free access programs. However, the cost of offering such programs may be recovered by raising the price of the drugs for paying patients and providers. Furthermore, some companies take advantage of orphan drug legislation by taking existing medicines through the path laid out for orphan drug development, re-branding them as drugs for rare diseases and, once designated as orphan drugs, marketing them at an elevated price. One way of addressing this issue would be to institute a system to ensure transparency of the cost of production and fair pricing – although we were made aware that this issue is an extremely sensitive area in practice and hence any effort to introduce such a system would require careful planning and consultation with relevant stakeholders.

## 2. Clearer and simpler regulatory and assessment processes for orphan drugs are needed

The complexity of regulatory and HTA procedures was identified as a possible major reason why rare disease patients often face prolonged delays before drugs of proven efficacy become available. In Europe, all orphan drugs have to be approved first by the EMA, then through a different HTA system in each member state before they become available for patients, just like any other drugs. In England and Wales, the National Institute for Health and Care Excellence (NICE) is responsible for undertaking HTA, with the aim of ensuring equity in healthcare provision by the NHS. However, NICE’s technology assessment scheme is designed primarily for medicines targeting common diseases. NICE has also developed a separate scheme for Highly Specialized Technology, but this focuses on so-called ultra-rare diseases, while NICE’s administrative capacity to run this scheme since its re-organization seems rather limited. For most orphan drugs, a decision on whether to make them available to patients is left to the NHS, but presently no clear and transparent way of reaching this decision seems to exist.

Increasingly, the importance of early involvement of all relevant stakeholders in development of drugs for rare diseases is being stressed. The EMA and COMP now encourage companies to engage at an early stage both with them and with patients, in order to understand what kind of evidence of risks and benefits is required for obtaining market authorization, and to design effective methods for collecting such evidence among small and often widely scattered populations of rare disease patients. A similar emphasis is visible in pilot efforts to coordinate post-market health technology assessment and access processes across member states, for instance EUnetHTA and the Mechanism of Coordinated Access to orphan medicinal products (MoCA). However, while these efforts could potentially simplify the approval process and reduce the obstacles that prolong waiting times for patients, concerns remain about their implications for national decisions on health care provision and about potential inconsistencies – particularly between regulators and HTA bodies – of risk-benefit assessment for market and post-market authorization processes. Moreover, the politics of regulation and regulatory reform are complex and contentious, while the option of engaging in that political arena may entail very considerable opportunity costs, particularly to small organizations with limited resources.

## 3. The opportunity costs for patient organizations need to be recognized

Patient organizations are not homogeneous. Some organizations have a longer history than others, and some have access to more resources – in terms of both finance and expertise – than others. Most patient organizations deploy their resources across a range of arenas, addressing various aspects of the life of patients, including increasing awareness about their disease, collecting and disseminating information about adequate care, funding research projects and advocating for better health services. Identification of a potentially effective medication can lead patient organizations to divert substantial resources to support development of the drug in order to make it available for their patients as quickly as possible – but this often means sacrificing other important activities. The longer the drug development process takes, the more significant such opportunity costs for patient organizations will be. Moreover, while umbrella organizations like EURORDIS and the Genetic Alliance UK provide support to smaller patient organizations, not all organizations are equally capable of bearing the cost.

While patient organizations make every effort to reduce this impact on their overall capacity, those efforts may be hindered by lack of clarity in regulatory or commissioning procedures, or by sudden changes in those procedures. For instance, such changes may disrupt efforts to build on the experience of other organizations, particularly in gathering the information necessary to secure approval for or access to new medicines. Participants also reported cases where a patient organization was advised to fill out the wrong forms or had to wait longer than promised for a response from the regulatory authority or healthcare provider. Such disruptions greatly add to the opportunity costs to patient organizations, as well as prolonging waiting times for patients in urgent need of a drug. Much more care needs to be taken by regulatory authorities and healthcare providers to minimize these costs to patient organizations, both when administering established procedures and when introducing changes in procedure.

## 4. The role of patient-private partnerships in drug development needs to be more clearly specified

It is patients who know best what it is like to live with their disease or condition. That knowledge is invaluable, and is mobilized at many different stages of drug development by different stakeholders – for example when patient organizations support and fund the development of registries and natural history databases to manage and provide information about their members, both deceased and living, to help academics or companies design and shape their research projects. Such active partnerships with companies often proceed without problems in the pre-competitive stages of research and development, but may run into problems in the later stages of drug development. For instance, tensions may arise when more than one company becomes interested in conducting clinical trials of a drug for a very small patient population. A concern was also expressed about the risk of patient organizations associating too closely with partner companies, particularly where a company provides financial support to the patient organization or lists the organization as an advisor or consultant, as such links could potentially be seen as entailing a conflict of interest when a patient organization is asked to provide evidence for efficacy of the drug for approval or commissioning purposes.

Patient organizations are increasingly considered as valuable sources of evidence throughout the process of drug development, but questions about how that evidence is produced and valued remain underexplored. Regulators, HTA bodies and healthcare providers often solicit evidence based on patients’ personal experiences that ‘brings to life’ the disease or the condition that a drug is to treat. Some patient organizations have developed skills in crafting and delivering such evidence, while pharmaceutical companies also collect patients’ personal testimonies and submit them as a part of their new drug applications along with more conventional clinical trials data on the clinical benefits and risks of a drug. However, the precise role of such evidence in regulatory and healthcare decision making, and the value of such evidence compared to clinical trials data, remains unclear.

Some companies – mostly small- and medium-sized enterprises – have been better at working with patient organizations than others, and moves are under way in the United States to improve the quality of such interactions across the industry. In this context, it is critical to understand what counts as a good interaction and how it would benefit patients. One suggestion made in the workshop was that a clearer definition of the contributions that patient organizations can make to drug development might include defining an appropriate legal mechanism to determine what kinds of compensation such organizations are entitled to – either as individual organizations or as representatives of a larger patient community.

## 5. Discussions about rare diseases may need to reflect their changing relationship with more common diseases

In 2013, the British Government published the UK Strategy for Rare Diseases [23]. This Strategy resulted from intensive discussions among various stakeholders and hence covers a broad range of areas that affect the lives of rare disease patients. Yet implementation proceeds slowly, while the rare disease landscape continues to change and revisions may be required. In particular, the Strategy includes a commitment to develop a transparent process for introducing new therapies. But translating that commitment into an effective process has proved to be a challenge, and concerted effort by the NHS is urgently needed. Some workshop participants suggested that re-framing this commitment in relation to medicine more generally might help to make clear how important it is. One reason for commercial investment in the development of certain innovative medicines for rare diseases is because of expectations that those treatments, once successfully developed, would subsequently be applicable to other diseases. At the same time, genomic medicine is increasingly dividing common diseases into collections of rare diseases. Consequently, any effective provision for introducing new therapies would likely be more widely applicable than just to what are currently seen as rare diseases.

One approach that has already been implemented, and that looks to offer a promising way of sharing risks among key stakeholders and broadening accountability for drug development, is the use of so-called Managed Access Programmes in the United Kingdom. In such a program, patients are given access to a new drug as part of a post-marketing data collection effort, subject to pre-determined start and stop criteria agreed by all the stakeholders including clinicians, the manufacturer, a patient organization, NICE and the NHS. Access is promised for a pre-specified timeframe provided participating patients adhere to the treatment protocol and are judged to derive benefit from it. The data thus collected are evaluated by NICE after this pre-specified period when a final approval decision is taken. A similar approach has been adopted for allocation of the Cancer Drugs Funds at the NHS, suggesting that expansion of the scope of such programs to include both common and rare diseases may be valuable.

## 6. The impact of the Brexit decision needs to be assessed and necessary measures taken

The workshop took place only a few days after the result of the referendum on UK membership in the European Union was announced, and concern about the impact of the Brexit decision on rare diseases surfaced repeatedly throughout the proceedings. In Europe, rare diseases became recognized as a public health issue in the 1990s, primarily in relation to integration of European pharmaceutical market [7]. EU policies on rare diseases since then have had significant influence on the policy decisions of its member states, as exemplified by the UK Strategy for Rare Diseases, which was produced to comply with the 2009 recommendation of the Council of the European Union [24]. The Brexit decision may potentially impact on the regulation of medicines, on pharmaceutical and biotechnology companies, and on practical arrangements for research and clinical collaboration – all of which relate, in one way or another, to the issues discussed at this workshop. The decision raises a host of uncertainties which need to be monitored and appropriate and timely measures taken, particularly where negative impacts are expected.

## Conclusions

Although rare disease patient organizations strive to secure access to the drugs that patients need, the resources available to them to do so are limited. Consequently, in order to achieve economies and synergies, it is important that patient organizations work as far as possible in partnership, not just with one another, but also with other stakeholders, including companies, regulators, healthcare professionals, politicians and academics. However, it became apparent in the course of this workshop that partnership working can entail risks as well as benefits.

“Partnership” can mean different arrangements to different stakeholders – not all partners are equal, and some may be better positioned to define the terms of partnership than others. Rare diseases patients may be in a particularly weak position because of the small size of their community. Some patient organizations may afford to invest their resources in making the partnership work, but it should be recognized that they are the exceptions, rather than the rule. The issues identified in the workshop reflected the facts that partnership – be it with private companies or with regulatory agencies or HTA bodies – often involves compromise and that establishing an effective and mutually beneficial partnership can be a significant challenge to many. Also, as one participant pointed out during the workshop, partnership is not an answer in itself – that is, it does not necessarily lead to access to medicines. While good intentions and mutual respect are valuable, the ‘rightness’ of any partnership therefore needs to be assessed in terms of whether it helps to attain that goal.

The policy-engagement workshop format provided a valuable setting in which to explore these issues. Most workshop participants were familiar with at least some of these issues from their past experiences, but had not necessarily recognized them as systemic issues needing to be addressed at the societal level. In a field populated by many, relatively small organizations, there is often a tendency to adopt a local, particularistic view of the challenges one faces, and to regard success or failure as a matter of the particular skills, resources and opportunities each organization is able to mobilize – as a matter of how well one can ‘play the game.’ The workshop provided an opportunity for the participants to share their experiences in a safe space, and to get a more systematic view of how the particular challenges they face are framed and structured – how, in other words, the ‘rules of the game’ are themselves determined, and what sorts of measures may be needed to revise those rules in ways more favorable to the needs of rare disease patients.

This report presents some of the main observations to emerge from that workshop. It focuses on issues where a degree of common ground was established, in the expectation that this will provide a basis for further debate and action, not confined to the individuals and organizations that participated in the workshop itself. We did not undertake a formal evaluation of the impact of the policy-engagement workshop on the participants, chiefly because the process of engagement was expected to continue with the drafting, redrafting and approval of this report. However, we take the continued involvement of the participants in the production of this report, and their willingness to see it published, as an indication that, at minimum, the workshop provided an opportunity to air and publicize certain issues that they consider worthy of wider attention. In particular, we note that while the workshop focused on challenges encountered specifically in relation to rare diseases, the consensus among the participants was that the issues identified will also become relevant to more common diseases, particularly due to advances in genomic technology. Consequently, the dialogue started at the workshop needs to be expanded, not just to involve other rare disease patient organizations, but to wider patient communities – including in particular the growing community of rare cancer organizations. Given the design of our workshop, we have no intention of claiming that it offers an answer to the complex problems and challenges that it explored, We would hope, however, that the workshop, and this report, might mark the beginning, not the end, of an expanding process of multi-stakeholder discussion and action around the problems of securing access to treatment to a growing number of rare diseases.

## Abbreviations

COMP: The Committee for Orphan Medicinal Products
EMA: The European Medicines Agency
HTA: Health technology assessment
MoCA: The Mechanism of Coordinated Access to orphan medicinal products
NHS: The National Health Service
NICE: The National Institute for Health and Care Excellence
